# Composition and potential anticancer activities of essential oils obtained from myrrh and frankincense

**DOI:** 10.3892/ol.2013.1520

**Published:** 2013-08-08

**Authors:** YINGLI CHEN, CHUNLAN ZHOU, ZHENDAN GE, YUFA LIU, YUMING LIU, WEIYI FENG, SEN LI, GUOYOU CHEN, TAIMING WEI

**Affiliations:** 1 College of Pharmacy, Harbin Medical University-Daqing, Daqing, Heilongjiang 163319, P.R. China; 2 Biopharmaceutical Institute of the Heilongjiang Academy of Medical Sciences, Harbin, Heilongjiang 158000, P.R. China; 3 Department of Chemistry, Chemical Engineering and Material Science, Shandong Normal University, Jinan, Shandong 250000, P.R. China; 4 College of Chemistry and Chemical Engineering, Tianjin University of Technology, Tianjin 300000, P.R. China; 5 College of Medicine, Xi'an Jiaotong University, Xi'an, Shanxi 710001, P.R. China

**Keywords:** myrrh, frankincense, essential oil, gas chromatography mass spectrometry, antiproliferative activity, apoptosis

## Abstract

The present study aimed to investigate the composition and potential anticancer activities of essential oils obtained from two species, myrrh and frankincense, by hydrodistillation. Using gas chromatography-mass spectrometry (GC-MS), 76 and 99 components were identified in the myrrh and frankincense essential oils, respectively, with the most abundant components, 2-Cyclohexen-1-one, 4-ethynyl-4-hydroxy-3,5,5-trimethyl- and *n*-Octylacetate, accounting for 12.01 and 34.66%, respectively. The effects of the two essential oils, independently and as a mixture, on five tumor cell lines, MCF-7, HS-1, HepG2, HeLa and A549, were investigated using the MTT assay. The results indicated that the MCF-7 and HS-1 cell lines showed increased sensitivity to the myrrh and frankincense essential oils compared with the remaining cell lines. In addition, the anticancer effects of myrrh were markedly increased compared with those of frankincense, however, no significant synergistic effects were identified. The flow cytometry results indicated that apoptosis may be a major contributor to the biological efficacy of MCF-7 cells.

## Introduction

*Commiphora myrrha* has a yellow oleo-gum resin that exists in its stem and is used worldwide for the production of myrrh, particularly in China and Egypt. The constituents of myrrh, include volatile oil (2–8%), resin (23–40%), gum (40–60%) and bitter principles (10–25%). Previous studies have shown that myrrh exhibits cytotoxic, analgesic, anti-inflammatory, anticancer, antiparasitic and hypolipidemic activities (1–4).

Frankincense is an aromatic resin obtained from trees of the genus *Boswellia* and has been hypothesized to exhibit a number of health supporting properties, including the treatment of rheumatoid arthritis and anti-inflammatory, antibacterial, antifungal and anticancer activities (5–8). Frankincense oil is prepared by the steam distillation of frankincense gum resin and is frequently used in aromatherapy practices. According to previous studies, the constituents of frankincense oil vary according to the climate, harvest conditions and geographical sources of the frankincense resin (9).

Notably, these two resinous drugs are always prescribed simultaneously in traditional Chinese medicine and are primarily administered for the treatment of blood stagnation and inflammation diseases, as well as for the relief of swelling and pain (10). A previous study identified that the combination of frankincense and myrrh oils exhibited synergistic effects on *Cryptococcus neoformans* and *Pseudomonas aeruginosa*(11).

The present study investigated the chemical composition of hydrodistilled frankincense and myrrh oils from Ethiopia. In addition, the anticancer activities of the prepared essential oils against the MCF-7, HepG2, HeLa, HS-1 and A549 cell lines were investigated to determine whether synergistic effects were observable *in vitro*. The results illustrated that certain cells (MCF-7 and HS-1 cells) demonstrate increased sensitivity to the two essential oils, and the anticancer effects of myrrh is superior to frankincense. No synergistic effect was observed.

## Materials and methods

### Materials

Dry sap samples were obtained in Ethiopia from the stem bark of *Boswellia carterii* and *Commiphora pyracanthoides* Engler in August 2009. The plant materials were identified by a botanist at Harbin Medicine University-Daqing (Daqing, China) and a voucher specimen was stored at the Department of Pharmacology (School of Pharmacy, Harbin Medicine University-Daqing).

### Extraction of essential oils

Subsequent to being frozen for 24 h, 30 g of the air-dried frankincense and myrrh samples were crushed into a powder. The essential oils from each sample were obtained through hydrodistillation for 3 h, according to the AB method described previously (12). Subsequently, the essential oils were diluted with 1% Tween 80 for a bioactivity analysis. The solution was prepared by mixing the myrrh and frankincense essential oils in a 1:1 ratio.

### GC-MS analysis

Analyses of the constituents of the essential oils were performed using gas chromatography mass spectrometry (GC-MS; Agilent Technologies, Santa Clara, CA, USA) and the GCMS-QP2010S mass spectrometer (Shimadzu Corp., Kyoto, Japan) with Rtx^®^-50 elastic quartz capillary column (30×0.25 mm, 0.25 μm) and helium carrier gas (Beijing AP BAIF Gases Industry Co., Ltd., Beijing, China). The injector temperature was 230°C and the interface and ion-source heating temperatures were 300°C and 230°C, respectively. The temperature program consisted of 60°C for 1 min and 220°C for 15 min, with a heating rate of 5°C/min. The column head pressure was 70 kPa, the EI-mode was 70 eV and the scan-range was 20–500 amu with a cycle time of 0.65 sec. Mass spectral correlations were performed using NIST05.

### Cell culture

Human cell lines (American Type Culture Collection, Rockville, MD, USA) obtained from breast (MCF-7) and hepatocellular (HepG2) carcinomas and cervical (HeLa), skin (HS-1) and small cell lung (A549) cancers, were maintained in monolayer tissue culture Petri dishes prior to examination. RPMI-1640 medium was supplemented with 10% fetal bovine serum (both Sigma-Aldrich, St. Louis, MO, USA), 100 IU/ml penicillin, 100 μg/ml streptomycin and 2 mM/l glutamine and cultures were maintained in a humidified atmosphere at 37°C in 5% CO_2_.

### MTT antiproliferative assay

3-(4,5-dimethylthiazol-2-yl)-2,5-diphenyltetrazolium bromide (MTT) method was used to determine the effects of frankincense and/or myrrh essential oils on cell proliferation in the MCF-7, HepG2, HeLa, HS-1 and A549 cell lines. Briefly, 5×10^3^ cells/well were evenly distributed and incubated on 96-well plates (Iwaki, Tokyo, Japan) overnight. The cells were then treated with frankincense, myrrh and a mixture of the essential oils at concentrations of 0, 5, 10, 20, 40, 60, 80, 160, 180, 320 and 640 μg/ml, and incubated for 24 and 48 h. Subsequently, the medium in each well was replaced with 20 μl MTT (5 mg/ml in PBS) and incubated at 37°C for 4 h. The purple-blue formazan precipitate was dissolved in 100 μl dimethyl sulfoxide and the optical density was measured at a wavelength of 570 nm on a 96-well plate reader (Thermo Labsystems, Franklin, MA USA). The IC_50_ was calculated as the concentration of compounds that achieved a 50% inhibition of cell viability. Data were analyzed using a SlideWrite program (Advanced Graphics Software, Inc., Rancho Santa Fe, CA, USA) to determine the IC_50_ of each drug independently.

### Synergistic effect analysis

Isobologram curves were derived as described previously (13): IC_50_ A and B = D_A_ / IC_X,A_ + D_B_ / IC_X,B_; where IC_50_ A and B indicates the combination concentration of drugs A and B at 50% inhibition, IC_X,A_ and IC_X,B_ indicates the concentration of the drugs that result in 50% inhibition independently and D_A_ and D_B_ indicates the concentrations of the two drugs as a mixture to achieve 50% inhibition. The isobologram curve was generated by plotting doses of drugs A vs. B predicted to simultaneously achieve 50% cell growth inhibition. A standard line of Loewe additivity was included to indicate a lack of interaction, and points below and above the line indicated synergy and antagonism, respectively.

### Cell apoptosis assay

Flow cytometry was used for the quantitative measurement of apoptosis. Briefly, 1×10^6^ MCF-7 cells were treated with 0, 10, 20 and 40 μg/ml frankincense and/or myrrh essential oils for 24 and 48 h, respectively. The cells were then collected by trypsinization and washed once with cold PBS. BD tubes were used and 100 μl suspension was added to each labeled tube followed by 10 μl Annexin V-FITC and 10 μl PI (20 μg/ml). Following incubation for ≥20 min at room temperature in the dark, 400 μl PBS binding buffer was added to each tube without washing. Within 30 min, the mixtures were analyzed using flow cytometry (BD FACSAria; BD Biosciences, San Jose, USA).

## Results

### GC-MS analysis

The content of the extracted oil of myrrh and frankincense was ~0.41 ml (2.05%, ml/g) and 0.62ml (2.06%, ml/g), respectively, and the total ion figures of the constituents were obtained by GC-MS analysis. The area normalization method was adopted to integrate the total ion peaks and the minimum area of the comparatively small peaks was set. Using a standard mass spectrum, 76 components were identified that accounted for 87.54% of the total myrrh essential oil (Table I). In addition, 99 components were identified that accounted for 91.26% of the total frankincense essential oil (Table II).

### MTT antiproliferative assay

Myrrh and frankincense essential oils exhibited an inhibitory effect on the cell lines and a dose-dependent inhibition effect was noted. Among the five cell lines, MCF-7 and HS-1 were sensitive to the myrrh and frankincense essential oils (Table III).

### Synergistic effect analyses

**Figure 1:**
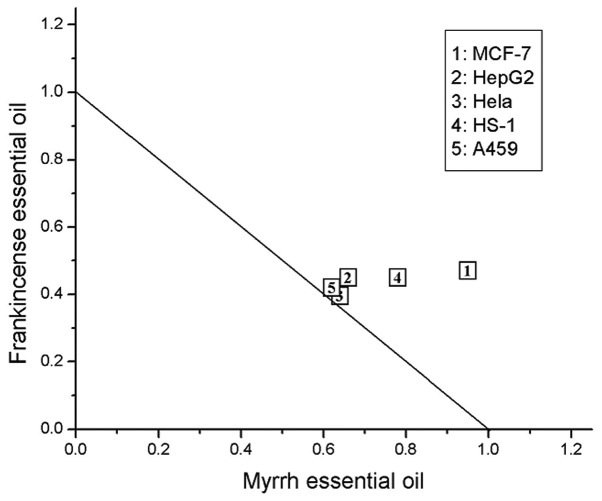
Isobologram and combination index curves at 50% effect level using combinations of myrrh and frankincense essential oils.

All points were identified above the standard line of Loewe additivity, therefore, no synergistic effects were identified in the isobologram and combination index curves (Fig. 1).

### Cell apoptosis assay

**Figure 2:**
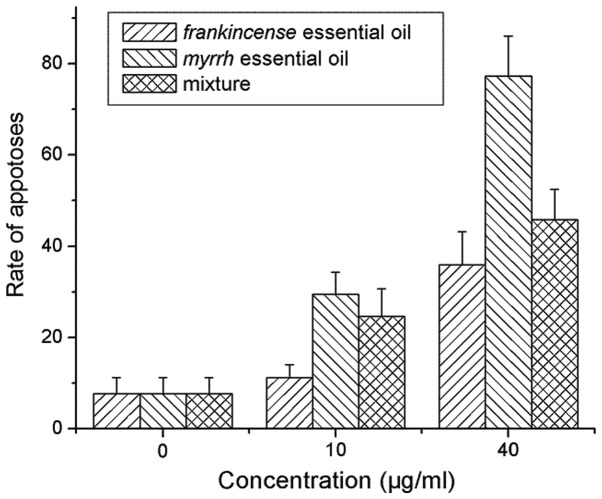
Flow cytometric analysis of myrrh, frankincense and the mixture of essential oils-induced apoptosis in the MCF-7 cell line following 24 h of treatment with 0, 10 and 40 μg/ml, respectively. Data are presented as the mean ± SD.

The flow cytometry results showed that the myrrh, frankincense and the mixture of essential oils were capable of inducing apoptosis in the MCF-7 cells in a concentration-dependent manner (Fig. 2). A dose-dependent induction of the apoptotic cells was performed to investigate the apoptosis rate. The early- and late-stage apoptosis rates of the MCF-7 cells induced by 40 μg/ml myrrh, frankincense and the mixture of essential oils were 36.0, 77.3 and 45.8%, respectively (P<0.01).

## Discussion

In the present study, the constituents of the essential oils of myrrh and frankincense were identified to include monoterpenes, sesquiterpenes, alcohols and esters. 2-Cyclohexen-1-one, 4-ethynyl-4-hydroxy-3,5,5-trimethyl was demonstrated to account for the highest percentage of the components in myrrh (12.01%), followed by β-elemene, copaene and aromadendrene, dehydro (6.18, 5.50 and 4.62%, respectively). By contrast, *n*-Octyl acetate was the most significant component of frankincense, accounting for 34.66%, followed by nerolidolisobutyrate, 3,7,11-trimethyl-1,6,10-dodecatrien-3-ylester-formic acid, δ-elemene and *n*-Octanol (18.29, 9.61, 5.61 and 3.24%, respectively). In contrast with the results of a previous study (14), additional components were detected in the frankincense oil, including β-elemene, α-pinene and *n*-Octanol (5.61, 0.07 and 3.24%, respectively).

A significant inhibitory effect was noted in the cell lines following treatment with the myrrh essential oil compared with treatment with frankincense and the mixture of essential oils. This observation indicated that apoptosis may be a major contributor to the biological efficacy of the MCF-7 cells. The apoptosis rate was higher in the myrrh essential oil group compared with that of the frankincense and mixture of essential oil groups at three concentrations (P<0.01). In addition, the results indicated that the breast cancer cell line exhibited increased sensitivity to the myrrh essential oil. To the best of our knowledge, the present study investigated the synergistic effects of the two drugs in the tumor cell lines for the first time. No synergistic effects were identified, which is in contrast to results observed using the Chinese folk formula (10).

Using cancer cell apoptosis induction trials, previous studies have identified that specific components of myrrh and frankincense essential oils are capable of inducing cancer cell apoptosis. For example, sesquiterpenes have anticancer activities that are likely to arrest the proliferation of prostate cancer cells in the G_0_/G_1_ phase (15–17). In addition, β-elemene has been reported to show pharmacological effects (18,19). In the present study, the IC_50_ of β-elemene in the MCF-7, HS-1, HepG2, HeLa and A549 cell lines was 14.7, 21.6, 16.1, 20.1 and 30.0 μg/ml (data not shown), respectively. Notably, the cell lines were more sensitive to β-elemene compared with frankincense and myrrh, indicating that β-elemene is important for the antitumor activity of the frankincense and myrrh essential oils. Previous studies have identified antitumour activity in two compounds with slightly greater contents of volatile oil, τ-cadinol, D-limonene, *n*-Octanol, δ-elemene, aromadendrene and (−)-Spathulenol (20–23). However, the activities and mechanisms of specific compositions must be investigated in future studies.

## Figures and Tables

**Table I:** Chemical composition of myrrh essential oil.

No.	Compound	RI^a^	%^b^
1	Bicyclo[2.2.1]heptane, 2-(1-methylethenyl)-	969	0.03
2	Azulene	1069	0.05
3	(+)-Cycloisosativene	1125	0.27
4	Acetic acid, octyl ester	1183	0.10
5	Ylangene	1221	0.10
6	Copaene	1221	5.50
7	5H-inden-5-one, 1,2,3,6,7,7a-hexahydro-7a-methyl-	1237	1.73
8	Seychellene	1275	0.57
9	Cyclohexane, 1,2-diethenyl-4-(1-methylethylidene)-, *cis-*	1281	0.36
10	Biurea	1328	0.02
11	β-bourbonene	1339	2.06
12	(+)-Sativen	1339	0.11
13	Isosativene	1339	0.02
14	α-cubebene	1344	0.39
15	δ-elemene	1377	2.51
16	7-Tetracyclo[6.2.1.0(3.8)0(3.9)]undecanol, 4,4,11,11-tetramethyl-	1385	0.03
17	Aromadendrene	1386	0.63
18	Tricyclo[6.3.0.0(2,4)]undec-8-ene, 3,3,7,11-tetramethyl-	1391	0.23
19	Aromadendrene, dehydro-	1396	4.62
20	β-elemene	1398	8.57
21	α-longipinene	1403	0.07
22	1,4-Diisopropyl-2,5-dimethylbenzene	1403	0.65
23	2-Cyclohexen-1-one, 4-ethynyl-4-hydroxy-3,5,5-trimethyl-	1406	12.00
24	1,1,4,7-Tetramethyl-1a,2,3,4,4a,5,6,7b-octahydro-1H-cyclopropa[e]azulene	1419	0.47
25	α-bergamotene	1430	0.70
26	*trans*-α-bergamotene	1430	0.93
27	Cyperene	1432	0.25
28	γ-muurolene	1435	0.78
29	Aminourea	1437	0.03
30	α-amorphene	1440	1.96
31	Naphthalene, 1,2,3,4,4a,7-hexahydro-1,6-dimethyl-4-(1-methylethyl)-	1440	0.13
32	β-panasinsene	1441	0.41
33	7-Oxabicyclo[4.1.0]heptane, 2,2,6-trimethyl-1-(3-methyl-1,3-butadienyl)-5-methylene-	1452	0.54
34	Bicyclo[5.3.0]decane, 2-methylene-5-(1-methylvinyl)-8-methyl-	1456	4.37
35	Aromadendrene oxide-(2)	1462	0.18
36	γ-elemene	1465	4.52
37	Nitrogen	1468	0.03
38	β-cadinene	1469	2.74
39	1-Cycloheptene, 1,4-dimethyl-3-(2-methyl-1-propene-1-yl)-4-vinyl-	1480	0.42
40	α-guaiene	1490	0.20
41	α-bulnesene	1490	1.17
42	4,11,11-Trimethyl-8-methylenebicyclo[7.2.0]undec-4-ene	1494	1.68
43	Humulen-(v1)	1494	0.36
44	2,4-Dimethyl-3-nitrobicyclo[3.2.1]octan-8-one	1498	0.20
45	Germacrene	1515	0.52
46	Germacrene D	1515	3.81
47	Cyclopropa[c,d]pentalene-1,3-dione, hexahydro-4-(2-methyl-2-propenyl)-2,2,4-trimethyl-	1518	0.35
48	Elemol	1522	3.96
49	4-(1-Methylethylidene)-1,2-divinylcyclohexane	1530	0.57
50	Epiglobulol	1530	0.27
51	Cyclononasiloxane, octadecamethyl-	1535	0.43
52	Ent-spathulenol	1536	3.34
53	(−)-Spathulenol	1536	0.32
54	3,7-Cyclodecadien-1-one, 10-(1-methylethenyl)-, (E,E)-	1562	2.00
55	Nerolidol	1564	0.04
56	Humulene	1579	0.80
57	τ-cadinol	1580	1.90
58	β-cadinol	1580	0.41
59	Longipinocarveol, *trans*-	1599	0.51
60	Azulen-2-ol, 1,4-dimethyl-7-(1-methylethyl)-	1602	0.78
61	Nickel tetracarbonyl	1623	0.02
62	6-Isopropenyl-4,8a-dimethyl-1,2,3,5,6,7,8,8a-octahydro-naphthalen-2-ol	1690	0.13
63	Cadalene	1706	0.16
64	2-(4a,8-Dimethyl-1,2,3,4,4a,5,6,7-octahydro-naphthalen-2-yl)-prop-2-en-1-ol	1745	0.25
65	3a,9b-Dimethyl-1,2,3a,4,5,9b-hexahydrocyclopenta[a]naphthalen-3-one	1747	0.06
66	Benzofuran, 2,3-dihydro-2-methyl-5-phenyl-	1763	0.07
67	Bicyclo[4.1.0]heptan-2-ol, 1β-(3-methyl-1,3-butadienyl)-2α,6β-dimethyl-3β-acetoxy-	1801	0.02
68	Nerolidol isobutyrate	1889	0.05
69	Dihexyl phthalate	1908	0.04
70	2(3H)-Naphthalenone, 4,4a,5,6,7,8-hexahydro-4-phenyl-	1918	1.89
71	N-(Trifluoroacetyl)-N,O,O′,O″-tetrakis(trimethylsilyl) norepinephrine	2151	0.89
72	Dinordesoxy-9-methyl-1, 8-diacetyleseroline	2152	0.22
73	4-Butylbenzoic acid, 2,7-dimethyloct-7-en-5-yn-4-yl ester	2223	0.22
74	Retinol acetate	2362	0.21
75	(4α,5α,17β)-3,17-dihydroxy-4,5-epoxyandrost-2-ene-2-carbonitrile	2427	0.24
76	(+)-Epi-bicyclosesquiphellandrene	2682	0.56

a Retention index;

b relative percentage obtained from peak area.

**Table II:** Chemical composition of frankincense essential oil.

No.	Compound	RI^a^	%^b^
1	α-pinene	948	0.07
2	Sabinene	897	0.02
3	Nopinene	943	0.02
4	β-myrcene	958	0.03
5	Octanal	1005	0.03
6	Hexyl acetate	984	0.10
7	*o*-Cymene	1024	0.03
8	D-Limonene	1018	0.30
9	Eucalyptol	1059	0.09
10	β-*trans*-ocimene	976	0.04
11	β-*cis*-ocimene	976	0.13
12	Tricyclene	998	0.01
13	*n*-Octanol	1059	3.27
14	β-linalool	1082	0.38
15	Nonanal	1104	0.02
16	1,3-Dimethylcyclohexene	852	0.58
17	L-pinocarveol	973	0.02
18	Isoborneol	1138	0.03
19	4-Terpineol	1137	0.07
20	Naphthalene	1231	0.09
21	3-Cyclohexene-1-methanol	1137	0.09
22	*n*-Octyl acetate	1183	34.66
23	*cis*-Geraniol	1128	0.03
24	*n*-Decanol	1158	0.09
25	1,7,7-Trimethylbicyclo[2.2.1]hept-2-yl acetate	1277	1.08
26	2-Dodecanone	1151	0.02
27	Octane	1042	0.03
28	*n*-Nonyl acetate	1282	0.03
29	Benzyl butyl ether	1264	0.02
30	(−)-Myrtenyl acetate	1314	0.04
31	Bornylene	1243	0.03
32	δ-elemene	1377	0.67
33	Citronellol acetate	1302	0.38
34	1,10-Decanediol	1356	0.04
35	Longicyclene	1184	0.07
36	Cubebene	1344	0.08
37	Nerol acetate	1352	0.82
38	Cyclobuta[1,2:3,4]dicyclopentene, decahydro-3a-methyl-6-methylene-1-(1-methylethyl)-, [1S-(1α,3aα,3bβ,6aβ,6bα)]-	1339	0.16
39	Decyl acetate	1381	0.72
40	1,4-Methanoazulene, decahydro-4,8,8-trimethyl-9-methylene-, (1S,3aR,4S,8aS)-	1398	0.40
41	Cyclopentane, 1-acetoxymethyl-3-isopropenyl-2-methyl-	1315	0.07
42	Caryophyllene oxide	1494	0.13
43	Bergamotol, Z-α-*trans*-	1673	0.05
44	Isoamyl caprylate	1364	0.03
45	(+)-Sativen	1339	0.05
46	Longicyclene	1184	0.09
47	Dodecanoic acid, 4-penten-1-yl ester	1281	0.04
48	α-humulene	1579	0.07
49	Hexahydrobenzylacetone	1440	0.03
50	α-Amorphene	1429	0.18
51	Germacrene	1515	0.76
52	(Z)-11-Tetradecen-1-ol acetate	1787	0.28
53	α-muurolene	1440	0.09
54	α-dodecene	1235	0.02
55	β-bisabolene	1500	0.06
56	γ-muurolene	1435	0.05
57	Methyl dodecanoate	1457	0.03
58	γ-cadinene	1469	0.12
59	Isophytol	1899	0.03
60	2-(4-Ethyl-4-methyl-3-(isopropenyl)cyclohexyl)propan-2-ol	1500	0.08
61	γ-elemene	1465	0.19
62	1,10-Decanediol	1501	0.11
63	Hexyl octanoate	1580	0.64
64	4-Camphenylbutan-2-one	1451	0.11
65	(−)-Spathulenol	1536	0.23
66	(−)-δ-cadinol	1420	0.09
67	(2E,6E,10E)-12-Hydroxy-3,7,11-trimethyl-2,6,10-dodecatrienyl acetate	2076	0.14
68	1-Pentadecanol	1543	0.05
69	Octyl heptanoate	1602	0.04
70	(Z)-11-Tetradecenyl acetate	1787	0.19
71	10-Isopropenyl-3,7-cyclodecadien-1-one	1745	0.06
72	Octanoic acid, phenylmethyl ester	1756	0.05
73	Octanoic acid, octyl ester	1779	0.32
74	2,4a,5,6,7,8,9,9a-octahydro-3,5,5-trimethyl-9-methylene-1H-benzocycloheptene	1826	0.04
75	Farnesyl acetate	1834	0.06
76	Lanceol, *cis*	1737	0.07
77	Cycloheptane, 4-methylene-1-methyl-2-(2-methyl-1-propen-1-yl)-1-vinyl-	1541	0.82
78	Cembrene	1687	0.24
79	Alloaromadendrene oxide-(2)	1435	0.30
80	β-elemene	1398	5.61
81	6-Isopropenyl-4,8a-dimethyl-1,2,3,5,6,7,8,8a-octahydro-naphthalen-2-ol	1690	0.14
82	Isophyllocladene	1794	0.73
83	Methyl (4Z,7Z,10Z,13Z,16Z,19Z)-4,7,10,13,16,19-docosahexaenoate	2523	0.17
84	Elixene	1431	2.30
85	Verticiol	2190	1.25
86	α-guaiene	1523	0.51
87	Thunbergol	2211	0.49
88	3-Ethyl-3-hydroxyandrostan-17-one	1953	0.19
89	α-santalol	1454	0.26
90	Epiglobulol	1530	0.17
91	Globulol	1530	0.12
92	α-bulnesene	1438	0.10
93	Formic acid, 3,7,11-trimethyl-1,6,10-dodecatrien-3-yl ester	1752	9.61
94	Nerolidol isobutyrate	1889	18.30
95	Cycloartanyl acetate	2956	0.05
96	(2,2,6-Trimethylbicyclo[4.1.0]hept-1-yl)-methanol	1673	0.02
97	Allopregnane-7α,11α-diol-3,20-dione	1794	0.04
98	Nerolidol isobutyrate	1889	0.33
99	4,8,13-Duvatriene-1,3-diol	1891	0.04

a Retention index,

b relative percentage obtained from peak area.

**Table III:** IC_50_ of myrrh, frankincense and the mixture of essential oils on the MCF-7, HepG2, HeLa, HS-1 and A549 cells at 24 h.

	Cell line IC_50_, μg/ml				
	
Essential oil	MCF-7	HepG2	Hela	HS-1	A459
Myrrh	19.8	39.2	34.3	22.7	41.4
Frankincense	40.7	57.0	55.5	39.7	60.3
Mixture, 1:1	38.1	51.4	43.9	35.4	51.0
